# Primary Cardiac Myxofibrosarcoma: A Case Report

**DOI:** 10.70352/scrj.cr.24-0035

**Published:** 2025-05-01

**Authors:** Takuya Matsushiro, Tomoki Tamura, Tetsuya Horai, Kento Misumi, Nobuyuki Inoue

**Affiliations:** 1 Department of Cardiovascular Surgery, National Center for Global Health and Medicine, Shinjuku, Tokyo, Japan; 2 Division of Pathology, Laboratory Testing Department, National Center for Global Health and Medicine, Shinjuku, Tokyo, Japan

**Keywords:** primary cardiac myxofibrosarcoma, recurrent cardiac myxoma, tumor incarceration

## Abstract

**INTRODUCTION:**

Primary cardiac tumors are rare, with malignant myxofibrosarcoma cases being particularly uncommon, while cardiac myxoma is a common diagnosis in clinical practice. We present a case of cardiac myxofibrosarcoma, whose clinical course was quite unusual.

**CASE PRESENTATION:**

A 67-year-old woman with no prior history of cardiac tumors was admitted for dyspnea and was found to have a 60 mm tumor in the left atrium. Despite initial resection, the tumor recurred twice, and a subsequent pleural mass biopsy revealed metastasis of myxofibrosarcoma. Retrospective analysis confirmed that the pathological findings of all the resected tumors were consistent with myxofibrosarcoma. Chemotherapy with doxorubicin was initiated, but severe side effects led to its discontinuation. The tumors continued to grow, causing significant pain, and she passed away a year later.

**CONCLUSIONS:**

The differential diagnosis of cardiac myxofibrosarcoma is challenging due to its similarity to benign myxomas. Accurate diagnosis requires thorough histological and immunohistochemical evaluation is essential.

## Abbreviations

CT
: computed tomography
ED
: emergency department
OR
: operating room

## INTRODUCTION

Most primary cardiac tumors are benign. When identifying a pedunculated tumor in the left atrium, cardiac myxoma is usually considered as the diagnosis. However, although primary cardiac malignancy is rare, it does exist, and thus a differential diagnosis is necessary. Myxofibrosarcoma is one of the possible primary cardiac malignancies. This tumor is rare, and a poor prognosis is predicted. Its high mortality rate is due to a high rate of recurrence. Complete surgical removal followed by chemotherapy is considered the standard treatment.^1)^ Meanwhile, an effective chemotherapy regimen for cardiac myxofibrosarcoma has not been established. In this report, we present a case of a patient with primary cardiac myxofibrosarcoma whose clinical course was quite unusual.

## CASE PRESENTATION

**Fig. 1:**
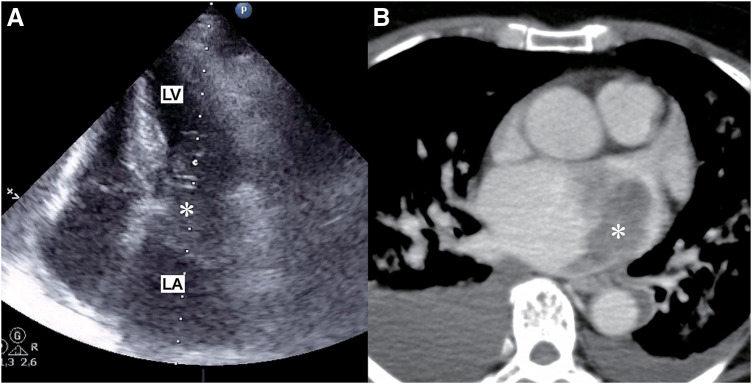
Echocardiography and CT scans. The echocardiogram (**A**) and contrast-enhanced CT radiograph (**B**) acquired upon the initial arrival of the patient show that a large tumor (asterisk) in the left atrium was incarcerated in the mitral annulus. CT, computed tomography; LA, left atrium; LV, left ventricle

**Fig. 2:**
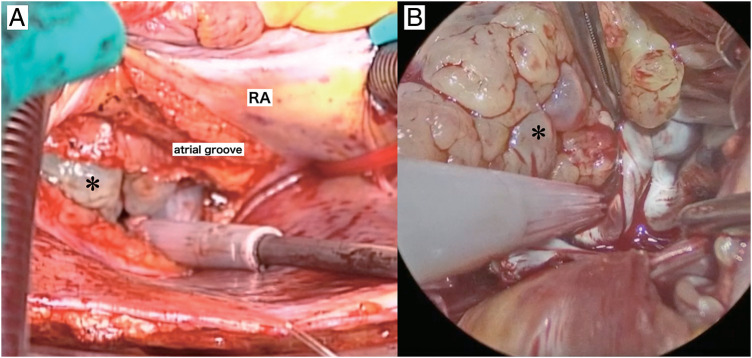
Endoscopic and operative photographs. In the operative photograph (**A**), a myxomatous tumor (asterisk) is visible in the left atrium, and the endoscopic view during surgery (**B**) shows that the tumor (asterisk) was completely detached from the atrial wall. RA, right atrium

**Fig. 3:**
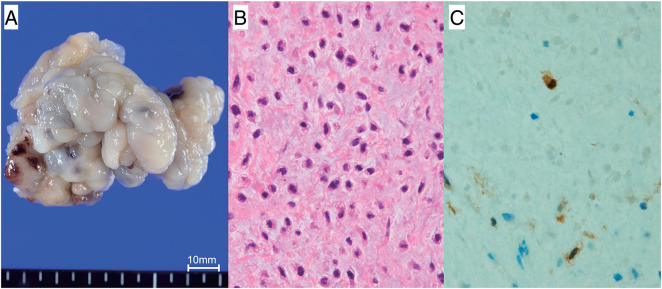
Histopathologic findings of the specimen resected during the first surgery. A myxomatous tumor shown in the macroscopic view was approximately 60 mm in size (**A**). Histopathologic analysis of the surgical specimen revealed low nuclear atypia and scarce mitotic figures (**B**, ×400 magnification); however, immunohistochemical results showed that the surgical specimen was almost negative for calretinin (**C**, ×400 magnification).

**Fig. 4:**
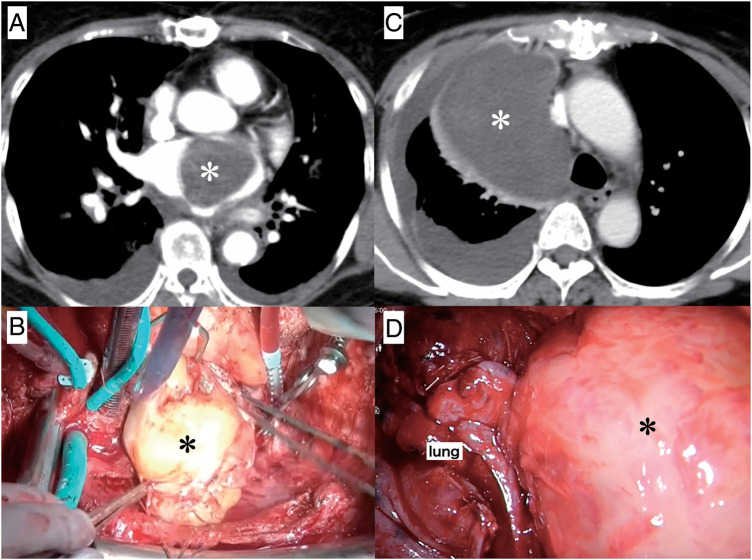
Follow-up tomography results. The CT radiographs acquired 2 years after the first surgery (**A**) revealed tumor (asterisk) recurrence. The operative photograph taken during redo surgery (**B**) shows a myxomatous tumor (asterisk), similar to the first one, being removed from the left atrium. The CT radiograph acquired 5 months after the last surgery (**C**) showed a tumor (asterisk) in the right pleural cavity. The thoracoscopic image (**D**) showed a large tumor (asterisk) adhering to the lung. CT, computed tomography; IVC, inferior vena cava; SVC, superior vena cava

**Fig. 5:**
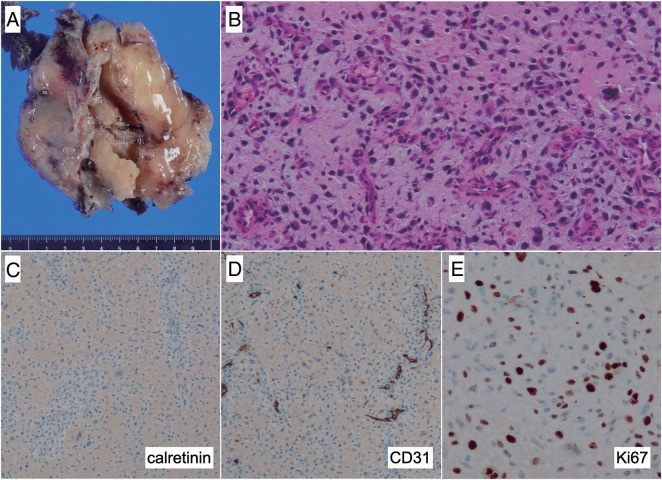
Histopathologic findings of the specimen resected from the pleural cavity during the last surgery. A large solid tumor (**A**) is visible in the macroscopic view. Histologic micrographs revealed eosinophilic cytoplasm, nuclear atypia, and mitotic figures (**B**, ×200 magnification). The surgical specimen was negative for calretinin (**C**, ×100 magnification), while being positive for CD31 (**D**, ×100 magnification) and Ki-67 (**E**, ×400 magnification).

A 67-year-old woman was transferred to the emergency department (ED) in our hospital and presented with dyspnea that had lasted for several days. She did not have any medical history or family history of cardiac tumors. Coarse crackles were heard, and her chest X-ray revealed pulmonary edema. An echocardiogram and contrast-enhanced computed tomography (CT) showed that the cause of the acute heart failure was a 60 mm diameter tumor in the left atrium that had become lodged in the mitral valve (**Fig. 1**). Clinically, the tumor was unequivocally diagnosed as a simple myxoma. She was taken to the operating room (OR). Cardiopulmonary bypass was established via bicaval drainage returning into the ascending aorta. The tumor was resected through right-sided left atriotomy (**Fig. 2**). It originates from the center of the posterior wall of the left atrium, between the left and right pulmonary veins. The tumor, along with its site of origin, was completely resected. The defect in the posterior atrial wall was repaired with a bovine pericardial patch. Pathological examination revealed a tumor with myxoid stroma. The tumor was found to have exhibited mild nuclear atypia and was negative for calretinin. No destructive invasion was observed. Although some features were uncommon, the tumor was still considered to fall within the myxoma (**Fig. 3**). 7 months later, during her regular check-up, a CT angiography showed that the left atrial tumor had recurred. The tumor was completely resected using a transseptal approach. The origin of the tumor was seen to be adjacent to the orifice of the left atrial appendage, near the patch sewn onto the posterior wall of the left atrium. The pathological features were similar to those reported previously. She had been followed up by a cardiologist after the second surgery and was admitted to our ED 16 months later. She presented with cardiogenic shock and dyspnea due to acute heart failure. The ensuing echocardiogram and CT angiography showed a large recurrent tumor in the left atrium that had become incarcerated in the mitral valve (**Fig. 4A**). She was rushed to the OR, and the tumor was resected from the left atrium again (**Fig. 4B**), with the same pathological findings. 5 months later, her chest radiograph showed a massive low-density lesion in the right pleural space (**Fig. 4C**). When punctured, the pleural effusion could not be aspirated. An excisional biopsy of the pleural mass was conducted via a small intercostal incision. The biopsy revealed more pronounced nuclear pleomorphism and mitoses. The patient tested negative for calretinin. The Ki-67 index was 50%, indicating that the tumor was not a myxoma. Considering the clinical course, previous pathological diagnoses were also reviewed, and the tumor was initially diagnosed as myxofibrosarcoma that had metastasized to the pleural space. 1 week later, she underwent video-assisted tumor resection via thoracotomy (**Fig. 4D**). Because the tumor adhered to the lung during surgery, a portion of the lung was also removed along with the tumor. The resected tumor was myxomatous, similar to the previously resected ones (**Fig. 5A**). Immunohistochemical testing revealed the same findings as those of the preceding biopsy (**Fig. 5B**–**5E**). After being discharged, our patient was referred to the department of oncology. 2 months later, her CT scan showed a recurrence of tumors in the thoracic cavity and left atrium. At that time, she received doxorubicin at a dose of at 60 mg/m^2^. Her electrocardiogram showed atrial fibrillation, and echocardiography indicated an ejection fraction of 30%. Since starting chemotherapy, she had exhibited severe nausea, which resulted in the cessation of treatment. The tumors enlarged and caused her significant pain, and she passed away a year later.

## DISCUSSION

Primary cardiac tumors occur with a frequency of 0.3%–0.7%, and 25% of them are malignant.^2)^ Among malignant cardiac tumors, angiosarcoma is the most common, while myxofibrosarcoma has been reported to account for 3.3%.^3)^ Like other malignant cardiac tumors, a myxofibrosarcoma tumor expands asymptomatically. The presenting symptoms are often due to heart failure, including dyspnea, palpitation, and chest pain. In the patient described here, the initial surgery was performed due to acute heart failure and cardiogenic shock caused by a tumor that had become incarcerated in the mitral valve.

Although there have been several case reports in the past regarding cardiac myxofibrosarcoma existing,^4)^ no treatment guidelines have been established. As reported in the literature, most patients undergo resection surgery, followed in some cases by chemotherapy or radiation therapy. However, the prognosis is poor, especially for tumors with a diameter larger than 40 mm.^1)^ One of the reasons for the poor prognosis is the lack of effective chemotherapy. Currently, complete surgical removal of the tumor is considered the most effective treatment. However, depending on the location of the tumor, it may not be possible to secure adequate surgical margin, and this may lead to recurrence.

The gross appearance of primary myxofibrosarcoma is primarily solid and myxomatous, resembling that of a benign myxoma: gelatinous, multilobulated, nodular, or papillary. Histologically, compared to benign myxoma, cardiac myxofibrosarcoma cells exhibit a spindle or stellate shape in a myxoid interstice with curvilinear vessels. The cytoplasm is usually eosinophilic, elongated, and scant. The nucleus is often hyperchromatic and pleomorphic. Immunohistochemical staining is considered beneficial to confirm the diagnosis; however, there are no specific markers to identify a cardiac myxofibrosarcoma. Most cases are positive for vimentin and occasionally positive for CD34, Ki-67, calponin, SMA, and CD117. On the other hand, cytokeratin, CD31, EMA, and S-100 are usually negative.^1)^ The difference between myxoma and myxofibrosarcoma is that the former frequently shows calretinin positivity, while the latter exhibits nuclear atypia.

In the present case, the resected mass macroscopically resembled a myxoma, and based on histopathologic findings, there was no doubt that it was a benign myxoma. However, the tumor recurred in a relatively short period, raising suspicions of malignancy, although histopathologic examination continued to support the diagnosis of benign myxoma. Despite its rarity, the recurrence of myxomas is possible,^5)^ and thus the present case was diagnosed as a recurrent myxoma. However, in benign cardiac myxoma, the recurrence interval is usually longer than 2 years.^1)^ Tumors that recur in the short term are usually considered malignant. When malignancy is suspected, an immunohistochemical examination should be conducted to arrive at a definitive diagnosis. In some reports, a cardiac myxofibrosarcoma in the left atrium has occasionally been mistaken for a cardiac myxoma.^6)^ Another piece of literature reported a case of myxofibrosarcoma misdiagnosed as a myxoma,^7)^ similar to our case. The differential diagnosis of these tumors is essential but challenging.

## CONCLUSIONS

The definitive diagnosis of myxofibrosarcoma of the heart is challenging because of its low incidence and similarities to benign myxomas. When the clinical presentation of an intracardiac tumor histologically diagnosed as a benign myxoma is unusual, it is necessary to consider myxofibrosarcoma as a possible differential diagnosis.

## DECLARATIONS

### Funding

Not applicable.

### Authors’ contributions

TM wrote the initial draft.

KM provided advice for pathological findings.

TT, NI, and TH supervised this presentation.

All authors have read and approved the final manuscript.

### Availability of data and materials

The datasets supporting the conclusions of this article are included within the article .

### Ethics approval and consent to participate

This work does not require ethical considerations or approval. Informed consent to participate in this study was obtained from the patients.

### Consent for publication

The patient provided informed consent for the publication of this report and accompanying images.

### Competing interests

The authors declare that they have no competing interests.

## References

[1] Sun D Wu Y Liu Y Primary cardiac myxofibrosarcoma: case report, literature review and pooled analysis. BMC Cancer 2018; 18: 512. 29720127 10.1186/s12885-018-4434-2 PMC5932848

[2] Bisel HF Wróblewski F LaDue JS. Incidence and clinical manifestations of cardiac metastases. J Am Med Assoc 1953; 153: 712–5. 13096280 10.1001/jama.1953.02940250018005

[3] Van Veer H Meuris B Verbeken E Primary atrial fibrosarcoma of the heart. Cardiovasc Pathol 2008; 17: 325–8. 18402789 10.1016/j.carpath.2007.04.010

[4] Soltani S Garousi M Mirzaee E A rare presentation of primary cardiac myxofibrosarcoma: case report and literature review. Cancer Rep (Hoboken) 2024; 7: e2033. 38600050 10.1002/cnr2.2033 PMC11006601

[5] Shinfeld A Katsumata T Westaby S. Recurrent cardiac myxoma: seeding or multifocal disease? Ann Thorac Surg 1998; 66: 285–8. 9692493 10.1016/s0003-4975(98)00481-0

[6] Reddy KVC Kumar P Sanzgiri P Primary cardiac myxofibrosarcoma with osteoid differentiation mimicking a left atrial myxoma: a rare entity. J Cardiol Cases 2020; 22: 253–6. 33133322 10.1016/j.jccase.2020.07.013 PMC7588476

[7] Das A Saha A Pattari S Myxofibrosarcoma or myxoma: malignant transformation or misdiagnosis. Indian J Thorac Cardiovasc Surg 2022; 38: 290–3. 10.1007/s12055-021-01298-8 PMC9023637 35529008

